# Bioactive Materials Promote Wound Healing through Modulation of Cell Behaviors

**DOI:** 10.1002/advs.202105152

**Published:** 2022-02-09

**Authors:** Ruotao Li, Kai Liu, Xu Huang, Di Li, Jianxun Ding, Bin Liu, Xuesi Chen

**Affiliations:** ^1^ Department of Hand and Foot Surgery The First Hospital of Jilin University 1 Xinmin Street Changchun 130065 P. R. China; ^2^ Key Laboratory of Polymer Ecomaterials Changchun Institute of Applied Chemistry Chinese Academy of Sciences 5625 Renmin Street Changchun 130022 P. R. China; ^3^ Department of Hepatobiliary and Pancreatic Surgery The First Hospital of Jilin University 1 Xinmin Street Changchun 130065 P. R. China

**Keywords:** bioactive material, cell behavior, wound microenvironment, wound healing, regenerative medicine

## Abstract

Skin wound repair is a multistage process involving multiple cellular and molecular interactions, which modulate the cell behaviors and dynamic remodeling of extracellular matrices to maximize regeneration and repair. Consequently, abnormalities in cell functions or pathways inevitably give rise to side effects, such as dysregulated inflammation, hyperplasia of nonmigratory epithelial cells, and lack of response to growth factors, which impedes angiogenesis and fibrosis. These issues may cause delayed wound healing or even non‐healing states. Current clinical therapeutic approaches are predominantly dedicated to preventing infections and alleviating topical symptoms rather than addressing the modulation of wound microenvironments to achieve targeted outcomes. Bioactive materials, relying on their chemical, physical, and biological properties or as carriers of bioactive substances, can affect wound microenvironments and promote wound healing at the molecular level. By addressing the mechanisms of wound healing from the perspective of cell behaviors, this review discusses how bioactive materials modulate the microenvironments and cell behaviors within the wounds during the stages of hemostasis, anti‐inflammation, tissue regeneration and deposition, and matrix remodeling. A deeper understanding of cell behaviors during wound healing is bound to promote the development of more targeted and efficient bioactive materials for clinical applications.

## Graphical abstract

Bioactive materials with specific chemical, physical, and biological properties or as the carriers of bioactive cargoes, regulate wound microenvironments and promote wound healing effectively, which work through modulation of cell behaviors and dynamic remodeling of extracellular matrices during the stages of hemostasis, anti‐inflammation, tissue regeneration and deposition, and matrix remodeling, providing an emerging modality for clinical wound therapy.

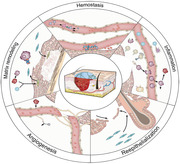

## 1 Introduction

Skin is the largest organ of the body, composed of various cell types that interact in a highly coordinated manner to maintain homeostasis.^[^ ^1^ ^]^ Cutaneous injuries, particularly chronic wounds, require long‐term treatment and place an enormous economic burden on healthcare systems. The prevalence of chronic wounds continues to increase with aging populations, coupled with rising rates of diabetes and obesity.^[^ ^2^ ^]^ Prolonged inflammation cycles, due to high protease levels, abnormal phenotypes of epidermis‐ and dermis‐derived cells, and biofilm colonization related to antibiotic resistance, may all contribute to the development of chronic wounds.^[^ ^3^ ^]^ Therefore, finding effective treatment options is crucial.

The wound microenvironments can be broadly divided into the external microenvironments directly adjacent to the wound surface and the internal conditions adjacent to the wound beneath the surface. The temperature, pH, water content, microbial content, partial pressure of oxygen (O_2_), and carbon dioxide (CO_2_) directly modify the external microenvironments and indirectly affect the internal microenvironments of the wound, such as cells and extracellular matrices (ECMs).^[^ ^3^ ^]^ An adequate understanding of the skin composition and healing mechanisms is crucial to achieving a directional impact on the wound microenvironments to accelerate healing.

The skin tissue consists of three main anatomical layers, namely, the epidermis, dermis, and subcutaneous tissue.^[^ ^1^ ^]^ The epidermis is the external, impermeable layer that is able to withstand the harsh conditions applied to the skin. It comprises five different layers, namely, basal, sphenoid, granular, hyaline, and stratum corneum. The dermis, located below the epidermis, is composed of papillary as well as reticular layers, and has an abundance of mechanoreceptors, ECMs, and blood vessels to provide nutrition, strength, and immunity for the skin. The subcutaneous tissue lies beneath the dermis as a continuous supply of growth factors (GFs) and plays a role in the energy reserve.^[^ ^2^, ^4^ ^]^

Several partially overlapping phases occur in a temporal sequence during cutaneous wound healing: hemostasis, inflammation, new tissue formation, and tissue remodeling. As one of the most complicated biological processes in the human body, wound healing involves regulating a sophisticated set of cell behaviors. The biological events involved in these phases are concisely controlled, including chemotaxis, phagocytosis, neocollagenesis, as well as collagen (Col) degradation and remodeling.^[^ ^5^ ^]^

In most cases, bleeding occurs immediately after the injury. The blood flushes out bacteria and other pathogens from the wound in the process of three fundamental procedures:^[^ ^6^ ^]^ vasoconstriction, primary hemostasis, and secondary hemostasis. The pivotal cell and matrix component involved in this process are the platelets and fibrinogen, respectively.^[^ ^2^ ^]^ After the injury, vasoconstriction of the vessel wall immediately responds as the first step in the hemostatic process. Subsequently, primary and secondary hemostasis function through two simultaneous and intertwined pathways.^[^ ^7^ ^]^ Eventually, the combination of fibrin mesh and platelet plug forms the thrombus to stop bleeding and stabilize the homeostasis. This creates a temporary scaffold that allows cell infiltration, which is required for healing.^[^ ^8^ ^]^

Simultaneously, the aggregated platelets secrete various cytokines and GFs that recruit neutrophils and monocytes to the wound site, initiating the inflammation stage of wound repair.^[^ ^9^ ^]^ Within minutes after the injury, neutrophils arrive at the wound as the first line of defense against pathogens, such as bacteria, viruses, and so forth.^[^ ^10^ ^]^ The release of chemotactic substances, such as chemokines (CC and CXC subfamilies) and some cytokines (tumor necrosis factor‐*α* (TNF‐*α*), interleukin‐1 (IL‐1), and interleukin‐6 (IL‐6)), amplifies the subsequent immune responses. Next, monocytes are recruited and transformed into macrophages to continue the cleansing process. The GFs released by macrophages over this period, such as transforming growth factors *α* and *β* (TGF‐*α*/*β*) and insulin‐like growth factor 1 (IGF‐1), are of paramount importance for the coordination and regulation of all ensuing phases of wound healing.^[^ ^4^ ^]^ Furthermore, the adaptive immune system, based on T cells, dermal dendritic cells, and Langerhans cells, is activated to fight against endogenous and exogenous antigens.^[^ ^11^ ^]^

Because of the attraction of factors secreted by macrophages and fibrin clots, fibroblasts are the first to reach the wound site after which the proliferation phase begins. This occurs 48−72 h after the migration of epidermal stem cells and basal keratinocytes,^[^ ^12^ ^]^ and involves several significant events, including reepithelialization, angiogenesis, and the formation of granulation tissue.^[^ ^13^ ^]^

Reepithelialization depends on the migration and proliferation of epithelial keratinocytes.^[^ ^14^ ^]^ The proliferation of cells involves both the proliferation of a basement membrane of unipotent epidermal stem cells and the dedifferentiation of epidermal cells that are already terminally differentiated.^[^ ^15^ ^]^ Keratinocytes located in the specific microenvironments facilitate the release of segregated GFs, such as TGF‐*β* and epidermal growth factors (EGFs), from the impaired ECMs.^[^ ^16^ ^]^ This mediates other indispensable aspects of tissue proliferation, like intercellular adhesion and reestablishing cell adhesion to the basement membrane.^[^ ^16c^ ^]^

Angiogenesis is defined as the growth of new blood vessels from previously existing vessels.^[^ ^17^ ^]^ The four main phases of angiogenesis include stimulation and rupture of the basement membrane, sprouting, tube formation, and maturation.^[^ ^18^ ^]^ The interactions between proangiogenesis factors and various cell receptors activate endothelial cells (ECs) from a quiescent state to a migratory and proliferative subtype. Meanwhile, endothelial cells produce matrix metalloproteinases (MMPs), playing an essential role in angiogenesis.^[^ ^19^ ^]^ ECs proliferate accompanied by the activation of pericytes in the basal layer, which helps build scaffolds and maintain their structural integrity.^[^ ^20^ ^]^

Pericytes, fibroblasts, smooth muscle cells, and ECs form the wound granulation tissue,^[^ ^21^ ^]^ which acts as a scaffold for other components and cells, like newly synthesized ECMs, blood vessels, and inflammatory cells. After the coordinated wound remodeling phase, granulation tissue is ultimately replaced by normal connective tissue.^[^ ^22^ ^]^ The formation of granulation tissue facilitates the regeneration of skin tissue by reestablishing the blood supply and the functional and mechanical integrity of connective tissue.^[^ ^16c^ ^]^

Stages of remodeling involve the degeneration of neovasculature, periodic deposition of new tissue into the ECMs, and reconstruction of granulation tissue to scar tissue.^[^ ^2^ ^]^ Fibroblasts in granulation tissue differentiate into myofibroblasts with a higher contractile capacity owing to mechanical tension, integrin signaling, and cytokines (e.g., TGF‐*β* and platelet‐derived growth factor (PDGF)). Myofibroblasts promote wound contraction, thus enhancing the mechanical strength of scar.^[^ ^23^ ^]^ Concurrent with the contraction, myofibroblasts synthesize *α*‐smooth muscle actin (*α*‐SMA) and MMPs, along with their respective inhibitors (tissue inhibitors of metalloproteinases, TIMPs). In this case, type III collagen (Col III) is replaced by type I collagen (Col I) with a more powerful tensile strength, yet the healed skin only reaches up to 80% of its original tensile strength.^[^ ^4^, ^24^ ^]^ During matrix remodeling, immune cells, such as macrophages and T lymphocytes, perform crucial phagocytosis of ECM debris and apoptotic cells and decompose excessive ECMs.^[^ ^2^ ^]^ The process described above can be considered as the complete process of skin repair.

Current clinical treatments, such as debridement, negative pressure wound therapy, interventional therapy, and topical antibiotics, only provide symptomatic treatment at the macroscopic level and do not actively ameliorate the wound microenvironments. Furthermore, as an essential component of skin tissue, ECMs are constantly remodeled to support cell and tissue morphogenesis rather than remaining in a quiescent role.^[^ ^25^ ^]^ The molecular composition and physical properties of ECMs determine the destiny and performance of cells while mediating intercellular communication through the transmission of mechanical signals.^[^ ^21b^ ^]^ Bioactive materials are, therefore, a viable therapeutic tool that mitigates the shortcomings of current strategies while influencing the wound microenvironments and inducing specific behaviors in pivotal cells, hence altering the healing process toward the desired direction.

**Scheme 1:**
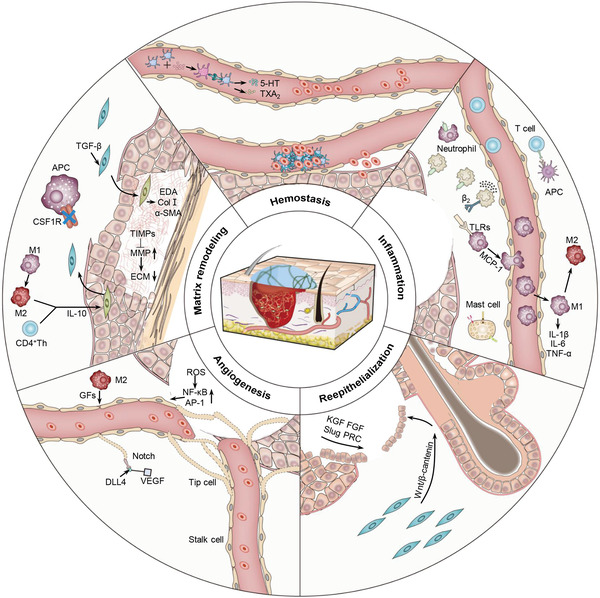
Schematic illustration of typical pathways of bioactive materials modulating cell behaviors to promote cutaneous wound healing.

In this review, the cellular and molecular mechanisms of each wound healing phase are elucidated in detail. The roles of bioactive materials in activating platelets, regulating the chemotaxis of immune cells, accelerating cell migration and proliferation, and controlling cell expression are elaborated (**Scheme** **1**). Finally, we present emerging opportunities and bottlenecks of bioactive materials for cutaneous wound healing.

## 2 Accelerated Wound Healing via Cell Behavior Modulation of Bioactive Materials

Cutaneous wound healing requires the coordination of several types of cells. Thus, approaches to modulate cell behaviors via bioactive materials are promising in the acceleration of skin wound healing, including facilitation of platelet activation and aggregation, regulation of immune cells chemotaxis and polarization, acceleration of cell proliferation and migration, as well as the control of cell expression. Mechanisms of different bioactive materials regulating cutaneous wound healing in various stages are summarized in **Table** **1**.

**Table 1:** Mechanisms of different biomaterials regulating cutaneous wound healing in different stages (Abbreviations: 3D, three‐dimensional; A‐MSC, adipose‐derived mesenchymal stem cell; Ag NP, silver nanoparticle; APC, antigen‐presenting cell; Au NP, gold nanoparticle; bFGF, basic fibroblast growth factor; BG, bioglass; BM‐MSC, bone marrow‐derived mesenchymal stem cell; CM, conditioned medium; Col, collagen; CPO, calcium peroxide; CSF1R, colony‐stimulating factor‐1 receptor; D‐MSC, dermal‐derived mesenchymal stem cell; DNA, deoxyribonucleic acid; EMT, epithelial‐mesenchymal transition; EndMT, endothelial‐mesenchymal transition; GAG, glycosaminoglycan; GF, growth factor; IL, interleukin; MA, methacryloyloxy; MB, microbubble; MRGPRX2, Mas‐related G‐protein coupled receptor member X2; MMP, matrix metalloproteinase; MNGC, multinucleated giant cell; NB, norbornenes; NOCS, *N*,*O*‐carboxymethyl chitosan; PAR‐1, protease‐activated receptors‐1; PCL, poly(ε‐caprolactone); PCN, polyethylenimine functionalized ceria nanocluster; PDGF, platelet‐derived growth factor; PDMS, polydimethylsiloxane; PEG, poly(ethylene glycol); PFD, pirfenidone; PLGA, poly(lactic‐*co*‐glycolic acid); PNIPAM, poly (*N*‐isopropyl acrylamide); PUAO, polyurethane; PVA, poly(vinyl alcohol); PVC, poly(vinyl chloride); ROS, reactive oxygen species; SA, sodium alginate; sHA3, high‐sulfated hyaluronic acid; TGF, transforming growth factor; TiO_2_ NP, titanium oxide nanoparticle; TLR, toll‐like receptor; TNF, tumor necrosis factor; TRAP‐6, thrombin receptor agonist peptide‐6; VEGF, vascular endothelial growth factor; ZnO NP, zinc oxide nanoparticle)

Stage	Bioactive materials	Type of cell or protein	Mechanism	Wound model	Refs.
Hemostasis	Photopolymerized PVA−NB hydrogel particle with TRAP6	Platelets	TRAP6 could activate platelets and aggregation via PAR‐1	Coagulation model	^[^ ^30c^ ^]^
	Interpenetrating polymer network dry cryogel	Blood cells/platelets	Catechol group and dopamine could reinforce blood cell/platelet adhesion and activation	Liver trauma, liver incision, and liver cross incision models	^[^ ^35^ ^]^
	Quaternized carboxymethyl chitosan and organic rectorite nanocomposite	Blood cells	The positive charge on the chitosan surface could aggregate blood cells	Skin trauma model	^[^ ^38^ ^]^
Anti‐inflammation	Modular hydrogel consisted of GAG heparin derivatives and star‐shaped PEG	Neutrophils	The hydrogel could eliminate inflammatory chemokines	Chronic venous leg ulcer model	^[^ ^47^ ^]^
	Multilayer coating of heparin−chitosan	Neutrophils	It could downregulate the expression of *β*2 integrin and reduce neutrophil recruiting	–	^[^ ^49^ ^]^
	*β*‐sheet Q11 peptide grafted with glucomannan	Macrophages	Activate the mannose receptor to promote its polarization toward the M2 phenotype	Skin trauma model	^[^ ^55^ ^]^
	Negatively charged carboxylic acid‐terminated nanorod	Macrophages	Negative electricity could transform macrophages into an anti‐inflammatory M2 phenotype	–	^[^ ^56^ ^]^
	Stiff natural biopolymer matrices composed of Col I and GAGs	Macrophages	Macrophages demonstrated M2 phenotype on it	–	^[^ ^58^ ^]^
	sHA3 covalent binding to Col fibril	Macrophages	It reduced macrophage M1 response and did not induce MNGC formation	Skin trauma model	^[^ ^59^ ^]^
	3D Col I fibronectin network	Macrophages	It could induce macrophage tolerance	–	^[^ ^60^ ^]^
	Cationic gelatin, cationic dextran, polyethyleneimine, and polylysine	T cells	Cationic polymers could induce potent Th1 responses via IL‐12 secretion mediated by TLR‐4	–	^[^ ^64^ ^]^
	PLGA nanoparticle	T cells	The PLGA nanoparticle act as APCs to promote the proliferation of T cells	Melanoma model	^[^ ^65^ ^]^
	Self‐assembling peptide (RADA)_4_ bound with PAMP‐12 motif	Mast cells	PAMP‐12 could activate mast cells via the MRGPRX2 receptor	–	^[^ ^67^ ^]^
	PVC surface modified with CD47	Neutrophils	CD47 could reduce neutrophil recruitment and adhesion	–	^[^ ^74^ ^]^
Tissue regeneration and Col deposition	OxOBand encapsulated with A‐MSC‐derived exosomes	Keratinocytes	Exosomes accelerate the migration rate of keratinocytes	Diabetic ulcer model	^[^ ^86^ ^]^
	Engineered human A‐MSC‐derived exosomes	Fibroblasts	miR‐21‐5p could promote reepithelialization through the Wnt/*β*‐catenin pathway	Diabetic ulcer model	^[^ ^90^ ^]^
	Electrospinning nanofiber scaffold containing Nagelschmidtite	Epithelial cells	Nagelschmidtite could activate both the EMT and EndMT pathways	Diabetic ulcer model	^[^ ^92^ ^]^
	PCL/Col nanofibrous matrix coated with Col gel	Keratinocytes	It could affect the migration of keratinocytes, enhance the expression of MMP‐2 and ‐9, promote the deposition of laminin‐332, and activate integrin *β*1	–	^[^ ^93^ ^]^
	Human recombinant Col VII	Keratinocytes	Col VII could mediate adhesion between epidermis and dermis in human skin	Recessive dystrophic epidermolysis bullosa model	^[^ ^94^ ^]^
	Microstructured Col membrane	Keratinocytes	The differentiation of keratinocytes was enhanced under the mimic natural 3D structure	–	^[^ ^95^ ^]^
	Tetrahedral DNA nanostructure	Endothelial cells	The nanomaterial could enhance angiogenesis by upregulating Notch signals	–	^[^ ^102^ ^]^
	Bioactive material loaded with VEGF, PDGF, bFGF, and TGF	Endothelial cells	Different GFs could regulate endothelial cells for angiogenesis	–	^[^ ^104^, ^105^, ^106^, ^107^ ^]^
	Borosilicate cross‐linked with SF via MA group loaded with Cu^2+^	Endothelial cells	The HIF‐1*α* pathway was restored by interaction with Cu^2+^	Diabetic ulcer model	^[^ ^112^ ^]^
	Multireactive injectable catechol–Fe^3+^ coordinated hydrogel	Endothelial cells	It could eliminate ROS, thus promoting neovascularization	Burn wound model	^[^ ^115^ ^]^
	PUAO−CPO cryogel	Endothelial cells	It displayed an excess ROS and reduction of angiogenesis	Ischemic flap model	^[^ ^116^ ^]^
	ZnO NP, TiO_2_ NP, Ag NP, Au NP lanthanide metallide NP, graphene oxide, and carbon nanotube	Endothelial cells	NPs could induce the formation of ROS and boost endothelial cell migration and incipient tube formation.	–	^[^ ^117^, ^118^, ^119^, ^120^, ^121^, ^122^, ^123^ ^]^
	Bioglass and mesoporous silica nanosphere fabricated on nanofibrous membrane	Endothelial cells	It could release silicon ions and upregulate the expression of genes associated with angiogenesis and new tissue formation	–	^[^ ^100^ ^]^
	Hydrogel based on fayalite and NOCS	Endothelial cells	It could stimulate the GF secretion to promote angiogenesis	Diabetic ulcer model	^[^ ^124^ ^]^
	PCN‐miR/COL hydrogel	Endothelial cells	It could reduce ROS and generate functional neovascularization	Diabetic ulcer model	^[^ ^125^ ^]^
	PNIPAM fiber in PDMS mold	Endothelial cells	3D network could form vascular perfusion throughout the hydrogel implant	Ischemic hindlimb and skin trauma models	^[^ ^126^ ^]^
	Copper‐containing mesoporous glass NP	Endothelial cells	It could promote endothelial cell proliferation and angiogenesis	Infected skin model	^[^ ^128^ ^]^
	MB with hydrogel	Endothelial cells	It could promote O_2_ diffusion and accelerate wound healing	–	^[^ ^129^ ^]^
	Dual drug‐loaded bilayer nanofibrous sponge‐like 3D scaffold	Fibroblasts	It could promote fibroblast migration and potentiate Col synthesis	Silicone splint model	^[^ ^130^ ^]^
	Nonmulberry silk fibroin	Keratinocytes and fibroblasts	RGD peptide on it could increase recruitment and adhesion of keratinocytes and fibroblasts, which accelerate the granulation formation	Skin trauma model	^[^ ^131^ ^]^
Matrix remodeling	starPEG–heparin hydrogel introducing RGD peptide	Fibroblasts	It could achieve sustained release of TGF‐*β* to induce fibroblasts into myofibroblasts	–	^[^ ^133^ ^]^
	SF hydrogel	Fibroblasts	It could induce the expression of TNF‐*α* and CD163	Burn wound model	^[^ ^134^ ^]^
	Sulfated GAGs	MMP	It could inhibit the MMP‐1 and ‐2	–	^[^ ^140^ ^]^
	Silk‐fibroin/gelatin electrospun nanofibrous dressing with astragaloside IV	Myofibroblasts and inflammatory cells	It could reduce TGF‐*β*1 secretion and Col I/III ratios	Burn wound model	^[^ ^141^ ^]^
	PLA electrospun with IL‐10‐HA‐sol inside and IL‐10 outside	Fibroblasts and macrophages	Released IL‐10 and promoted macrophage polarization toward the M2c phenotype	Skin trauma model	^[^ ^144^ ^]^
	SA/BG‐SA_CM_‐PLGA_PFD_	Fibroblasts and immune cells	The system could regulate the inflammatory response, promote the formation of vascularized granulation tissue, and prevent fibrosis and scarring of regenerative skin	Diabetic ulcer model	^[^ ^147^ ^]^
	Bioactive material implantation and inhibiting CSF1R	Macrophages	Inhibition of CSF1R could inhibit fibrosis and improve biocompatibility	–	^[^ ^150^ ^]^
	Integra loaded with A‐MSCs and D‐MSCs	Macrophages	Hydrogel combined with stem cells could modulate macrophage polarization.	Skin trauma model	^[^ ^152^ ^]^

### 2.1 Hemostasis by Facilitated Aggregation and Activation of Platelets

The crucial functions of hemostasis are adhesion, activation, and accumulation of platelets, which are anuclear cells sprouting from megakaryocytes at the impaired vessel endothelium.^[^ ^2^, ^26^ ^]^ Platelets and small amounts of blood cells adhere to the inner subcutaneous Col during primary hemostasis. This rapid adhesion activates other platelets in the blood, leading to an irreversible aggregation.^[^ ^27^ ^]^ The activated platelets play an essential role in enhancing vasoconstriction and reinforcing the first step in hemostasis by releasing thromboxane A2 (TXA2) and 5‐hydroxytryptamine (5‐HT). Activated platelets combined with multiple coagulation factors promote the production of cell‐based thrombin. Thrombin kinase produces thrombin that catalyzes the transition of fibrinogen to fibrin with the aid of transglutaminase (FXIII), facilitating the coagulation process.^[^ ^28^ ^]^ In turn, the production of thrombin activates platelets to potentiate their activity, providing positive feedback to the hemostatic process.^[^ ^27^, ^29^ ^]^ Moreover, thrombin activates platelets primarily through the stimulation of protease‐activated receptors‐1 (PAR‐1), triggered by the proteolytic cleavage of a portion of the extracellular *N*‐terminal region of the PAR‐1 receptor.^[^ ^30^ ^]^

**Figure 1:**
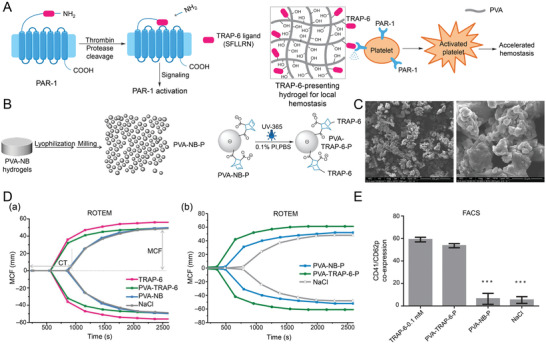
Synthetic platelet‐activating hydrogel to induce local hemostasis. A) Molecular mechanism of PAR‐1 activation and TRAP‐6‐presenting hydrogel. B) Preparation of PVA hydrogel (—SH: —NB = 0.4) particulate (PVA‐NB‐P) and surface functionalization of PVA‐NB‐P with cysteine‐containing TRAP‐6 peptide. C) SEM images of PVA‐TRAP‐6‐P. D) Plotted ROTEM curves show the coagulation process of whole blood in response to a) TRAP‐6, PVA‐TRAP‐6, PVA‐NB, and 0.9% NaCl as a control. b) PVA‐NB‐P and PVA‐TRAP‐6‐P suspensions, and 0.9% NaCl as a control. E) FACS analysis of TRAP‐6‐mediated platelet activation measured by determination of CD62p/CD41 coexpression. Reproduced with permission.^[^ ^30c^ ^]^ Copyright 2015, Wiley‐VCH. FACS, fluorescence‐ activated cell sorter; NB, norbornene; PAR‐1, protease‐activated receptors‐1; PVA, poly(vinyl alcohol); SEM, scanning electron microscopy; TRAP‐6, thrombin receptor agonist peptide‐6.

The complicated hemostasis cascade reaction provides numerous targets for developing bioactive materials to facilitate platelet activation and aggregation for skin wound healing. For example, a new *N*‐terminal ligand domain is generated by proteolysis, so‐called thrombin receptor agonist peptide‐6 (TRAP‐6), which triggers the activation of PAR‐1 by interacting with the receptors within the extracellular loop 2.^[^ ^30c^ ^]^ Qin et al. biofunctionalized the photopolymerized poly(vinyl alcohol)‐norbornenes hydrogel particle (PVA‐NB‐P) with TRAP‐6 to prepare PVA‐TRAP‐6‐P. Surprisingly, the PVA‐TRAP‐6‐P effectively reduced the clotting time by approximately 50% (**Figure** **1**). Platelet activity is also manipulated by some auto‐secreted significant granules, such as *α*‐granules.^[^ ^31^ ^]^ A study has shown that plasma fibronectin was rapidly released from *α*‐granule deposits on impaired vessel walls after bleeding, thus mediating a step that occurred even earlier than the adhesion and aggregation of platelets.^[^ ^32^ ^]^

**Figure 2:**
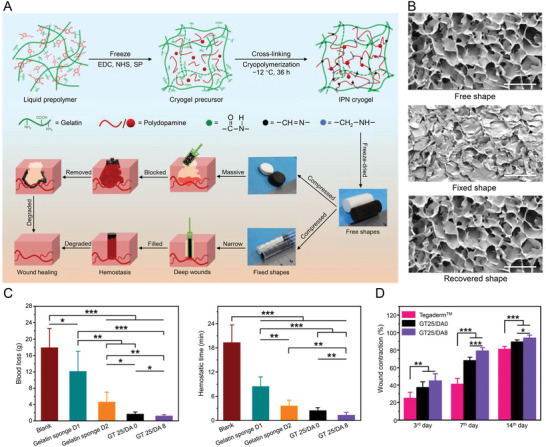
Degradable gelatin‐based IPN cryogel hemostat for rapidly stopping deep noncompressible hemorrhage and improving wound healing. A) Schematic representation of GT/DA cryogel formation and different fixed shapes of GT25/DA0 and GT25/DA8. B) Microtopography observation of GT25/DA8 in free shape, fixed shape, and recovered shape. Scale bar = 200 µm. C) In vivo hemostatic performance of cryogel in rabbit liver defect hemorrhage model. D) Wound contraction rate of in vivo wound‐healing evaluation. Reproduced with permission.^[^ ^35^ ^]^ Copyright 2020, American Chemical Society. DA, dopamine; EDC, 1‐(3‐dimethylaminopropyl)‐3‐ethylcarbodii‐mide hydrochloride; GT, gelatin; IPN, interpenetrating polymer network; NHS, *N*‐hydroxysuccinimide; SP, sodium periodate.

Apart from PAR‐1 and *α*‐granules, catechol group and dopamine demonstrate tissue adhesiveness and reinforce blood cell/platelet adhesion and activation.^[^ ^33^ ^]^ Hence, researchers have developed varying bioactive material containing catechol group or dopamine to enhance blood coagulation.^[^ ^34^ ^]^ For example, Huang et al. developed a hemostatic agent, a biodegradable interpenetrating polymer network (IPN) dry cryogel derived from the cryo‐polymerization of gelatin and dopamine (**Figure** **2**).^[^ ^35^ ^]^ Another experiment demonstrated its superior hemostatic property to medical gauze and commercially available PVA sponge and better wound healing results than Tegaderm film, a commercial product commonly used in clinical practice.

Another governing factor that regulates platelets due to the unique characteristics of blood cells is the surface charge. Blood cells will accumulate quickly when they contact positively charged surfaces. Because of the relatively large size and surface area, as well as morphological changes in the activated platelets, blood cells rapidly form blood clots responding to fibrin.^[^ ^36^ ^]^ Therefore, utilizing the surface charge of bioactive material to directly aggregate blood cells to participate in the coagulation system may be a viable method for hemostasis. Chitosan is the only edible fiber with positive charge on its surface, which shows excellent biocompatibility and contains amino, hydroxyl, and other polar groups. These properties make chitosan a proper choice for various biological applications. The healing bioactive material prepared by chitosan can reduce bleeding time by 90%.^[^ ^37^ ^]^ Li et al. designed an injectable nanocomposite by inserting quaternized carboxymethyl chitosan into the organic rectorite intermediate layer to form a physical cross‐linking network, which not only allowed natural clay to bind to tissues and blood cells through ionic interactions to achieve hemostasis but also prevented rectorite from entering the bloodstream to form thrombi.^[^ ^38^ ^]^

As the first step in wound healing, hemostasis is of critical importance. The activation of platelets and the release of factors enable subsequent healing steps, especially the inflammatory phase demonstrated below.

### 2.2 Anti‐Inflammation by Regulation of Chemotaxis and Polarization of Immune Cells

The injury primarily activates transcription‐independent pathways susceptible to stimulation, including purified molecules, reactive oxygen species (ROS) gradients, and Ca^2+^ waves.^[^ ^39^ ^]^ Furthermore, wounded cells can secrete damage‐associated molecular patterns (DAMPs, such as peptides, adenosine triphosphate, deoxyribonucleic acid (DNA), uric acid, and ECM components), lipid mediators, hydrogen peroxide (H_2_O_2_), and chemokines, likewise contributing to the recruitment of inflammatory cells, particularly neutrophils.^[^ ^40^ ^]^ Chemokines are small proteins containing cysteines as a part of the structure of which CC and CXC subfamilies are the most prominent. Most chemokine receptors bind multiple chemokines and subsequently trigger downstream pathways, leading to oriented cell movement or chemotaxis.^[^ ^41^ ^]^

**Figure 3:**
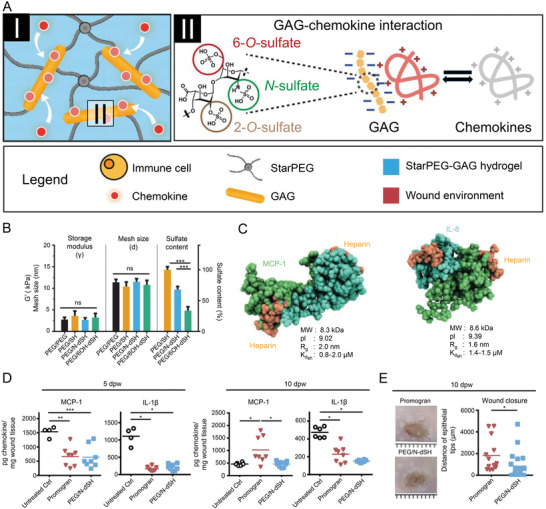
Glycosaminoglycan‐based hydrogel capture inflammatory chemokines and rescue defective wound healing. A) StarPEG‐GAG hydrogel network can bind and neutralize chemokines through strong electrostatic interactions of heparin derivatives and chemokines. B) Storage modulus, mesh size, and sulfate content of compared hydrogel. C) Results of computational docking analysis of both chemokines using ClusPro software. D) Characterization of inflammation 5 and 10 days after wounding. E) Wounds 10 dpw and analysis of wound closure. Reproduced with permission.^[^ ^47^ ^]^ Copyright 2017, American Association for the Advancement of Science. GAG, glycosaminoglycan; IL‐8, interleukin‐8; MCP‐1, monocyte chemoattractant protein‐1; PEG, poly(ethylene glycol).

Neutrophils are not commonly found in normal skin. They are recruited from the bone marrow to serve as the frontline of an inflammatory response.^[^ ^42^ ^]^ More than 30 different surface receptors assist neutrophils in detecting injury signals.^[^ ^43^ ^]^ After functionalization, the neutrophils can activate macrophage efferocytosis through matricellular protein CCN1 via the Rac1‐dependent pathway.^[^ ^44^ ^]^ Interestingly, the total elimination of neutrophils through efferocytosis marks the beginning of inflammation resolution. However, neutrophils do not seem to play a dominant role in cutaneous wound healing.^[^ ^45^ ^]^ In contrast, the persistent presence of neutrophils can lead to a long‐term inflammatory state and chronic wounds, such that their prompt removal is essential.^[^ ^46^ ^]^ Therefore, Lohmann et al. designed a modular hydrogel consisting of glycosaminoglycan (GAG) heparin derivatives and star‐shaped poly(ethylene glycol) (starPEG) to maximize the sequestration of chemokines (**Figure** **3**).^[^ ^47^ ^]^ This bioactive material almost eliminated inflammatory chemokines, such as macrophage inflammatory protein‐1, monocyte chemoattractant protein‐1 (MCP‐1), and IL‐8, from the wound fluid of chronic ulcer patients. Thus, it weakened the migratory activity of neutrophils and monocytes to the wound area.

Except for segregating chemokines, the *β*2 integrins on the surface of neutrophils, such as LFA‐1 and Mac‐1, are indispensable for the neutrophils' recruitment and site recognition to bioactive materials. After adhesion, activated neutrophils release myeloperoxidase, a ligand for Mac‐1, subsequently potentiating neutrophils' secretion of the pro‐inflammatory cytokines.^[^ ^48^ ^]^ Therefore, regulating their expression is a requisite for restricting neutrophil recruitment and reducing inflammation. Heparin exhibits blocking effects by occupying binding sites on receptors to prevent their activation.^[^ ^49^ ^]^ For example, a multilayer coating of heparin−chitosan effectively downregulated the expression of *β*2 integrin.

Thus far, the role of neutrophil recruitment and scavenging in wound healing is a relatively novel concern, and neutrophil regulation is still in the exploration stage. Since several studies have demonstrated its crucial role in wound healing, the subject deserves further attention. In time, bioactive materials modulating neutrophils to accelerate tissue regeneration and repair will motivate further research.

In humans, macrophages are generally identified by the expression of surface markers CD45^+^/Cd11b^+^/CD66b^−^.^[^ ^50^ ^]^ Both proliferation of local tissue‐resident macrophages and the recruitment of monocytes from the bone marrow can increase the macrophage quantity.^[^ ^2^ ^]^ As tissue‐resident macrophages are less informative in wound healing, more attention has been paid to monocytes, which can differentiate into macrophages in wounds.^[^ ^51^ ^]^ Such proinflammatory and bactericidal macrophages at the early stages of wound healing, secreting pro‐inflammatory factors, such as IL‐1*β*, IL‐6, and TNF‐*α*, known as M1 phenotype, phagocytose pathogens, and digest ECMs and thrombi.^[^ ^2^ ^]^ Through inflammasome signaling and toll‐like receptors (TLRs), the classical inflammation pathway is activated by pattern recognition receptors on macrophages that identify and bind to DAMP molecules.^[^ ^52^ ^]^ Downregulation of the transcription factor FOXM1 results in reduced infiltration of macrophages in diabetic ulcer and a decreased inflammatory response.^[^ ^53^ ^]^

As the inflammation subsides, the inflammatory phenotype of M1 macrophages shifts into an anti‐inflammatory phenotype known as the M2 phenotype, which can be categorized into three main subsets, including alternatively activated macrophages or profibrotic macrophages (M2a), immunoregulatory‐type macrophages (M2b), and fibrinolytic macrophages (M2c or M_reg_‐like cells).^[^ ^2^, ^54^ ^]^

Promoting macrophage transition to the M2 phenotype at a later stage of wound healing increases tissue regeneration efficiency. Feng et al. formed a glycopeptide hydrogel with *β*‐sheet Q11 peptide (QQKFQFQFEQQ) grafted with glucomannan, without the addition of any therapeutic agents to activate the primary macrophage mannose receptor via the ERK/STAT6 pathway and promote its polarization toward the M2 phenotype.^[^ ^55^ ^]^ This is because negatively charged carboxylic acid‐terminated nanorod induced an inflammatory M1 phenotype, although positively charged amine‐terminated nanorod transformed macrophages into an anti‐inflammatory M2 phenotype.^[^ ^56^ ^]^ Interestingly, the varying stiffness of bioactive materials affects the polarization of macrophages. A recent study found that the positive feedback between the cation channel Piezo1 and actin could promote the activation of macrophages, which demonstrated that Piezo1 acted as a mechanosensor of stiffness and was related to the polarization response.^[^ ^57^ ^]^ In particular, macrophages showed an M2 phenotype in stiffer natural biopolymer matrices composed of Col I and GAGs with increased secretion of IL‐10 and decreased secretion of IL‐12 and TNF‐*α*.^[^ ^58^ ^]^ Ghanaati et al. covalently bonded high‐sulfated hyaluronic acid (sHA3) to a Col scaffold, allowing for the marginal release of sHA3. This reduced the proportion of M1 macrophages and did not induce multinucleated giant cell (MNGC) formation within 30 days.^[^ ^59^ ^]^ However, our visions must not be merely limited to the polarization of M1 and M2 phenotypes, but focus on immune tolerance and training to reflect the diversity of macrophage phenotypes. Logie et al. discovered that a three‐dimensional (3D) Col I fibronectin network functionally induced macrophage tolerance by detecting the expression of *CLEC4E, SLC2A6, SOD2*, and *FABP4* genes.^[^ ^60^ ^]^

*αβ* ^+^ and *γδ* ^+^ T cells are the two T cell variants in the human's epidermis and dermis, where the former constitute the majority. *αβ* ^+^ T cells are composed of Foxp3^+^ T_reg_ subsets, CD4^+^ T helper cells (Th cells), and CD8^+^ T killer cells, which play as a memory function, either entering the blood circulation or permanently residing in the skin.^[^ ^2^, ^61^ ^]^ Antigens are presented to T cells residing in the dermis by dendritic cells, which migrate to the draining lymph nodes after this process and proceed with T cell response activation.^[^ ^62^ ^]^ Except for antigen presentation, skin fibrosis is likewise associated with T cell dysfunction. Hypertrophic scarring by burns in humans increases the number of T cells, while the higher mechanical forces likewise activate their pathways during the healing process.^[^ ^63^ ^]^

Cationic polymers, such as cationic gelatin, cationic dextran, polyethyleneimine, and polylysine, can induce potent Th1 responses via IL‐12 secretion mediated by TLR‐4 in vivo. The effect of the stimulus is determined by the polymer's molecular weight and the degree of cationization.^[^ ^64^ ^]^ Green's group applied poly(lactic‐*co*‐glycolic acid) (PLGA) nanoparticle as artificial antigen‐presenting cells (aAPCs) to simultaneously deliver antigen fragments and costimulatory markers to T cells. The ellipsoidal aAPCs stimulated more T cell proliferation than spherical ones owing to the increased contact with T cells and prolonged circulation time in mice.^[^ ^65^ ^]^

Mast cells act predominantly as allergic response effectors in the skin. They are activated directly by pathogens and antigens relying on Fc*ε*RI together with other pattern recognition receptors, e.g., rig‐like, nod‐like, toll‐like, and c‐type lectin.^[^ ^66^ ^]^ Increasing evidence suggests that elevated mast cell numbers are associated with skin fibrosis and scar formation. Although during diabetic ulcer healing, the reduction in mast cell quantity and degranulation has been demonstrated, distinct subsets of mast cells with various features may exist, which require identification.^[^ ^2^ ^]^ For example, the PAMP‐12 peptide can activate mast cells via the Mas‐related G‐protein coupled receptor member X2 (MRGPRX2) receptor. Through the glycine spacer group, Lu et al. bound the PAMP‐12 motif to the self‐assembling peptide (RADA)_4_ and mixed it with unmodified (RADA)_4_ to develop a nanofiber matrix.^[^ ^67^ ^]^ Interestingly, the mixing ratio could influence the activation of mast cells. Mast cells are challenging to mature in vitro and a limited number of cells can be isolated,^[^ ^68^ ^]^ the mechanism of mast cells' contribution to wound healing is yet to be investigated.

Despite the development of various bioactive materials for wound healing, the problem of biocompatibility is of significant concern. Unfortunately, the introduction of any foreign matter, including bioactive materials, can exacerbate foreign body response (FBR) by amplifying the inflammation degree at the injury site caused by the accumulation of innate immune cells, such as macrophages and neutrophils. In this circumstance, the inflammatory microenvironments can be an obstacle for bioactive materials to integrate with the surrounding native tissues.^[^ ^69^ ^]^ The surface modification of bioactive materials has been considered as an essential strategy in modulating the immune response. This is managed by adjusting the physicochemical and biological properties of the surface, including hydrophilicity, topography, surface charge, and protein adsorption.^[^ ^70^ ^]^ Although there is no clear evidence that specific chemical functional groups affect the behaviors of macrophages, research shows that the impact of bioactive materials on macrophages may be related to the thickness of the protein adsorbed. Materials that adsorb thicker protein layers lead to proinflammatory behavior, whereas the opposite ones lead to anti‐inflammatory behavior.^[^ ^71^ ^]^ The size of foreign matter is also one of the essential elements that affect FBR. Compared to implants with larger diameters, smaller ones tend to facilitate massive infiltration of pro‐inflammatory macrophages and neutrophils, generating fierce FBR and fibrosis. Hydrogels or alginate capsules, as well as plastics, metals, and ceramics with a diameter of 0.5 mm, were readily phagocytosed by neutrophils and encouraged macrophage recruitment, while the larger ones (≥1.5 mm) could eliminate FBR.^[^ ^72^ ^]^ When the bioactive material is immunologically inert or presents anti‐inflammatory qualities, the clearance of neutrophils and recruitment of macrophages are usually acute, permitting macrophages to switch from the M1 to the M2 phenotype.^[^ ^73^ ^]^ For instance, Finley et al. reduced neutrophil recruitment and adhesion by precoating modified CD47 on polyvinyl chloride surface.^[^ ^74^ ^]^ Numerous previously published review articles focused on the impact and application of surface modification techniques, providing preliminary knowledge for this comprehensive review.^[^ ^71^, ^75^ ^]^

Thus far, little is known about how the immunogenicity of the material itself can help tailor and manipulate the results of tissue engineering. Fortunately, the research fields of immunotherapy and vaccines have delivered worthy information on how material properties influence the immune response. This demonstrated that physicochemical attributes are capable of altering the immunogenicity of bioactive materials to design improved materials based on them, obtaining the desired immunological microenvironments.^[^ ^76^ ^]^

### 2.3 Tissue Regeneration and Collagen Deposition through Proliferation Acceleration and Migration of Cells

Multiple events are involved in the tissue regeneration and deposition phases, such as the formation of epidermal skin layer (reepithelialization), construction of blood vessels (angiogenesis), and temporary formation of ECMs (granulation tissue deposition), leading to wound contraction. This specific phase is regulated by delicate crosstalk among diverse cells, notably keratinocytes, fibroblasts, macrophages, and endothelial cells.^[^ ^77^ ^]^ The cellular mechanisms of these processes and how they can be inhibited and modified by bioactive materials are described below.

#### 2.3.1 Reepithelialization

The epidermis mainly consists of keratinocytes and is continuously updated by the proliferation and differentiation of stem cells. Terminal differentiation occurs once stem cells migrate upward from the basal layer to the surface, where they are expected to die and defluvium.^[^ ^78^ ^]^ A recent study suggested that reepithelialization of partial‐thickness wounds in humans occurs mainly in the progenitor cells from pilosebaceous units eccrine, sweat glands, and as a minor part, in the basal progenitor cells from the interfollicular epidermis. However, reepithelialization must originate from the interfollicular epidermal cells at wound edges in full‐thickness wounds, where skin appendages are disrupted.^[^ ^79^ ^]^

**Figure 4:**
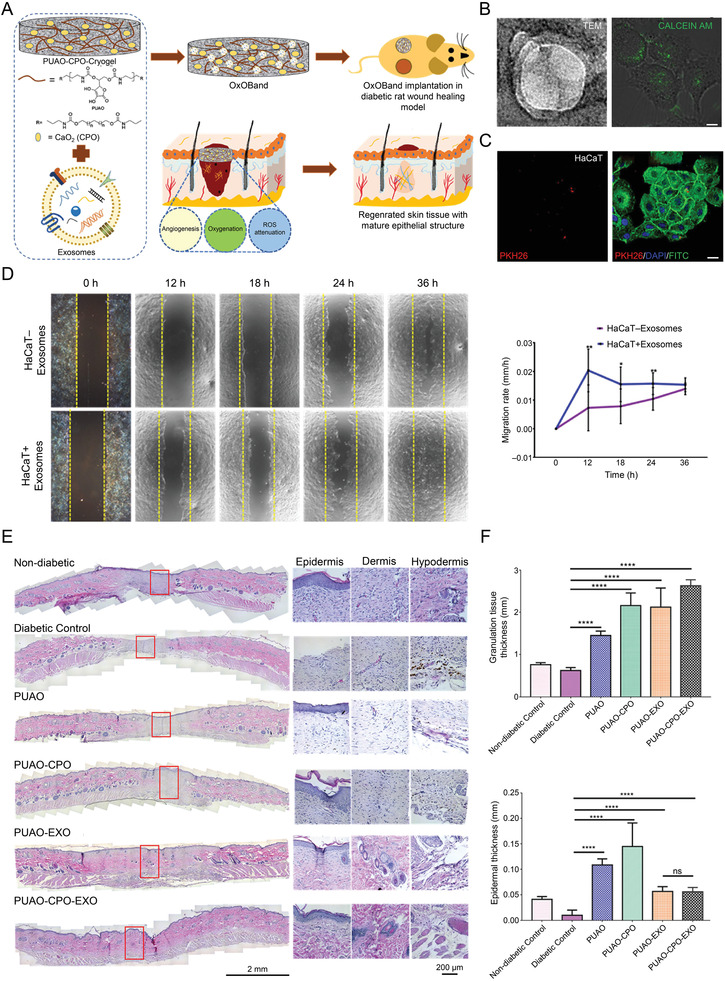
Exosome laden O_2_ releasing antioxidant and antibacterial cryogel wound dressing OxOBand alleviate diabetic and infectious wound healing. A) Schematic representation of OxOBand formation from O_2_ releasing antioxidant PUAO‐CPO cryogels with exosomes of A‐MSCs. B) (Left) Nanoparticle tracking analysis are showing the double membrane vesicular structure of the exosomes with cup‐shaped morphology. Scale bar = 200 nm. (Right) Calcein AM stained exosomes are showing the intact exosome structure encapsulated by HaCaT cells. Scale bar = 10 µm. C) Confocal laser scanning microscopic images showing encapsulation of PKH26 labeled exosomes (red) by HaCaT cells. Scale bar = 20 µm. D) HaCaT cell migration was enhanced by exosome treatment compared to nontreated cells. Representative graph showing the migration rate of HaCaT cells upon exosome treatment. E) Representative H&E images of wounds (scale bar = 2 mm) and high magnification images showing granulation tissue formation and epithelial tissue closure (scale bar = 200 µm). F) The granulation tissue formation and average epidermal thickness in the wound center were quantified in histological samples harvested after 14 days. Reproduced with permission.^[^ ^86^ ^]^ Copyright 2020, Elsevier Ltd. CPO, calcium peroxide; EXO, exosomes; H&E, hematoxylin and eosin; PUAO, polyurethane.

The migration of keratinocytes occurs during the early stage of wound reepithelialization for which different mechanisms have been proposed, such as rolling and sliding mechanisms.^[^ ^80^ ^]^ The phenomenon was demonstrated as mobile cohesive epithelial sheets at the anterior border migrating toward the wound center and experiencing fluctuations in cell membrane folds.^[^ ^81^ ^]^ As the epithelial cells migrate across the wound bed, adequate cell‐to‐cell adhesion is preserved, preventing further damage to the epithelial barrier. Recently, Costa et al. found that the directional migration of epithelial cells is mediated by calcium‐dependent cell−cell adhesion and microtubule networks by adopting a dynamically adhesive micropatterning platform.^[^ ^82^ ^]^ Understanding the crosstalk and dynamics of cell−ECMs and cell−cell interactions during the migration, alongside the mechanotransduction pathways engaged in pathological and physiological conditions, will assist in targeting the most suitable growth factors or adhesion substrates to add to bioactive materials. The intercellular adhesion will be attenuated, and their viability will be increased by several proteins and GFs, such as transcription factor Slug, protein kinase C‐*α* (PKC‐*α*), IL‐1, IL‐6, and TNF‐*α*.^[^ ^83^ ^]^ Keratinocytes respond to several factors from the epidermal growth factor family, such as TGF‐*β*, KGF, HB‐EGF, FGF, and EGF. Among these, KGF and FGF2 are crucial, as they upregulate the expression of keratin 6, 16, and 17, thereby promoting cell migration.^[^ ^84^ ^]^ Also, MMP‐9 is produced by migrating keratinocytes and helps degrade the dermal‐epidermal junction components for other keratinocytes to migrate throughout the wound.^[^ ^85^ ^]^ Kumar and co‐workers developed an antioxidant polyurethane (PUAO), OxOBand, encapsulated with adipose‐derived stem cell exosomes.^[^ ^86^ ^]^ Significant acceleration of the migration rate was observed after phagocytosis of exosomes by HaCaT keratinocytes. After 12 h of treatment, the migration rate was 0.020 ± 0.007 mm h^−^^1^, compared with 0.007 ± 0.0079 mm h^−1^ in the untreated group, indicating the accelerated wound reepithelialization process. Furthermore, they introduced the O_2_‐releasing PUAO‐calcium peroxide (CPO)‐EXO scaffold for experimental group. By quantifying the average epidermal thickness of wound center, it is reasonable to presume that both groups encapsulated with exosomes obtained higher maturation of epidermis (**Figure** **4**).^[^ ^86^ ^]^ When cutaneous damage occurs, the transepithelial cell potential of epithelial cells is disrupted, and the potential of wound rapidly decreases, creating a potential difference from that of undamaged tissue. Such potential gradients drive positive charge flow to the wound from surrounding tissues, thereby inducing directional cell migration.^[^ ^87^ ^]^ Thus, electroactive dressings providing electrical stimulation is likewise a promising strategy.

Approximately 2−3 days after injury, epidermal stem cells from the interfollicular epidermis on the wound margin, nearby sebaceous glands, or follicular bulges begin to proliferate to form sufficient cells to fill the wound site.^[^ ^80^ ^]^ The epidermal appendages constitute an integral part of the skin, which are exposed to external pressures and respond to skin damage. The hair follicles undergo a series of alternations in hair regeneration and degeneration, while the sweat glands are static.^[^ ^88^ ^]^ Unipotent stem cells in hair follicles migrate linearly and exhibit pluripotent differentiation potential to reestablish epidermal barriers containing multiple cells. Through the Wnt/*β*‐catenin signal pathway, fibroblasts in the dermal papilla send signals to stem cells in the hair follicle to activate the differentiation process.^[^ ^2^ ^]^ By utilizing this pathway, there have also been some relatively promising bioactive materials developed to induce reepithelialization. For example, EPS‐S3, an exopolysaccharide isolated from a kind of marine bacteria called *Pantoea sp* has been shown to regulate the healing process and promote reepithelialization through the Wnt/*β*‐catenin pathway, offering a candidate biomolecule for applications in cutaneous wound healing.^[^ ^89^ ^]^ Moreover, Lv et al. designed an engineered human adipose stem‐cell‐derived exosome delivering miR‐21‐5p to increase the reepithelialization and Col remodeling through the Wnt/*β*‐catenin pathway.^[^ ^90^ ^]^

Wound reepithelialization can be likened to a reversible and partial form of epithelial‐mesenchymal transition (EMT), a peculiar phenotypic alteration featuring the transformation of anchored epithelial cells into migrating fibroblast‐like cells. EMT includes the total dissociation of cell‐to‐cell adhesion structures, cell stretching, and reorganization of the cytoskeleton.^[^ ^80^ ^]^ The transcription factor Slug regulates several EMT‐like events in the process of reepithelialization.^[^ ^83b^ ^]^ Thus, even without the proliferation of keratinocytes, keratinocyte migration and Slug expression may also take place at the wound edges.^[^ ^91^ ^]^ A type of electrospinning nanofiber scaffold containing nagelschmidtite (NAGEL, Ca_7_P_2_Si_2_O_16_) has been biologically evaluated and shown to activate both the EMT and the endothelial‐mesenchymal transition (EndMT) pathways in vitro and in vivo.^[^ ^92^ ^]^

When adopting the bioactive material strategy, keratinocytes migrate from grafts or wound edges to bridge the opening wound for complete epithelial regeneration. Therefore, a microenvironment must be created to recruit keratinocytes and facilitate migration efficiently. By interacting with the cell receptor CD44, hyaluronic acid (HA) can modulate keratinocyte activities, which triggers specific signaling pathways resulting in migration and proliferation.^[^ ^107^, ^108^ ^]^ Coating Col gel on a polycaprolactone/Col nanofibrous matrix was found to have significant effects on the migration of keratinocytes and was shown to enhance the expression of MMP‐2 and ‐9, promote the deposition of laminin‐332 and activate integrin *β*1.^[^ ^93^ ^]^ Type VII collagen (Col VII) mediates adhesion between the epidermis and dermis in human skin. The application of human recombinant Col VII (rCol VII) to the grafts with recessive dystrophic epidermolysis bullosa (RDEB) may restore Col VII and anchor fibrils at the dermal–epidermal junction (DEJ), hence correcting dermal−epidermal separation.^[^ ^94^ ^]^ Microstructured Col membranes that mimic the natural 3D structure of the human skin papillary dermis may be an ambitious strategy to guide the behaviors of keratinocytes. The differentiation of keratinocytes was enhanced when cultured on microstructured substrates containing increased depth and decreased width micro‐channels that mimic the natural microenvironments of DEJ. Furthermore, facilitated reepithelialization was detected on the channels with the narrowest opening.^[^ ^95^ ^]^

A moist wound microenvironment is believed to accelerate reepithelialization, and the development of modern dressings offers a mild and moist condition for wound healing. At present, the dressings used for the clinical treatment of superficial wounds mainly include HA dressings, metalloproteinase inhibitors, hydrocolloid dressings, oil‐based dressings, interfaces, and hydrocolloids.^[^ ^80^ ^]^

Hyperkeratosis and parakeratosis are characteristics of the keratinocytes' abnormal presence in chronic wounds.^[^ ^96^ ^]^ Because of the activation and overexpression of *c‐myc*, keratinocytes at the unhealed edge of chronic wounds keep proliferating and dividing over the entire basal layer.^[^ ^97^ ^]^ In addition, the expression of integrins in suprabasal keratinocytes contributes to enhanced inflammatory cytokine and MAPK/Erk signaling synthesis, ultimately leading to immune cell activation and keratinocyte hyperproliferation.^[^ ^98^ ^]^ The loss of stem cell niche signaling in chronic venous ulcer results in hyperproliferation of the epidermis that exhibits the inability to migrate and close the wound and suffers from the aberrant differentiation of epidermal cells.^[^ ^99^ ^]^

The quality and rate of reepithelialization are generally related to the potential characteristics of ECM formation and angiogenesis. Further directions may be formulated to target specific cells to promote epidermal regeneration, preventing hyperkeratosis or parakeratosis. Future bioactive materials development efforts must focus on regulating the proper adhesion of ECM components to epithelial cells, allowing them to migrate and proliferate in an orderly manner.

#### 2.3.2 Angiogenesis

Angiogenesis involves a sequence of sophisticated and dynamic events that begins with stimulating ECs by proangiogenic factors. These factors result in their directed migration with the subsequent proliferation of adjacent cells and, ultimately, the formation of tubular structures.^[^ ^100^ ^]^ During angiogenesis, these cells can become either leading tip cells or trailing stalk cells whose developmental orientation is manipulated by Notch signaling, which is regulated by the vascular endothelial growth factor (VEGF) together with its effectors, Delta‐like 4 and Jagged 1.^[^ ^101^ ^]^ As an example, the tetrahedral DNA nanostructure is a new type of biocompatible nanomaterial that enhances angiogenesis by upregulating Notch signals,^[^ ^102^ ^]^ whose mechanism remains unclear to date. Tip cells extended their filopodia toward the increasing VEFG‐A gradient to control the vessel growth. In contrast, stalk cells, whose proliferation is VEGF‐A concentration‐dependent, tracked the tip cells and maintain the state of existing blood vessels, sprouting eventually into vascular tubules connected to other blood vessels by forming ECM signaling and new cell−cell junctions.^[2, 101a, 103]^ Therefore, loading GFs, such as VEGF,^[^ ^104^ ^]^ PDGF,^[^ ^105^ ^]^ basic fibroblast growth factor (bFGF),^[^ ^106^ ^]^ and TGF,^[^ ^107^ ^]^ onto or into bioactive materials is one of the most straightforward and frequently employed strategies to regulate endothelial cells for angiogenesis. To achieve goal of the controlled release of GFs, GFs and recombinant fibronectin fragments can be attached to the fibronectin scaffold, which also strengthens the interaction of GFs with the matrix and markedly reduces the concentration of morphogens required to generate effective tissue.^[^ ^108^ ^]^

Although ROS is commonly cytotoxic at high concentrations and can cause EC dysfunction, chronic inflammation, and oxidative stress, they can affect cell proliferation, migration, and differentiation at low concentrations. Interestingly, ROS at appropriate concentrations raise the expression levels of transcription factors NF‐*κ*B, activator protein‐1 (AP‐1), and E26 transformation specificity‐1 (ETS‐1) in endothelial cells and are capable of binding to the promoter of MMPs, such as collagenase and stromelysin.^[^ ^109^ ^]^ The induction of MMPs' activity is one of the initial features of angiogenesis, providing a provisional/interstitial matrix space for the migration of ECs.^[^ ^100^, ^110^ ^]^ Furthermore, by activating hypoxia‐inducible factor‐1*α* (HIF‐1*α*) and p38/MAPK/Akt pathways, ROS at low concentrations can induce appropriate cell signaling, contributing to the release of angiogenic factors.^[^ ^111^ ^]^ Pang et al. used borosilicates cross‐linked with silk fibroin (SF) via methacryloyloxy (MA) groups under ultraviolet (UV) radiation to form a hydrogel loaded with copper ions, showing that the HIF‐1*α* pathway was restored by interaction with Cu^2+^, and angiogenesis was enhanced as a result.^[^ ^112^ ^]^ In normal tissues, the steady‐state level of HIF‐1*α* is low, and it binds to the Von Hippel‐Lindau (VHL) tumor suppressor protein, forming a complex readily recognized and rapidly degraded by the proteasome.^[^ ^113^ ^]^ Hyperglycemia can also reduce the stability of HIF‐1*α*, leading to suppression of HIF‐1*α* target gene expression, resulting in delayed healing and ulcer complications in diabetic patients. According to the latest study, Li et al. synthesized a cyclometallated iridium(III) metal complex **1a** as a small molecule stabilizer of HIF‐1*α* that binds to VHL and blocks their interactions.^[^ ^114^ ^]^

Zhang et al. designed a multi‐reactive injectable catechol−Fe^3+^ coordinated hydrogel (MICH) matrix that exerts specific properties of “Fe‐superoxide dismutases” in mitigating cellular oxidative damage with precise elimination of ROS from different cell locations, preventing cell apoptosis and lipid peroxidation, thus promoting neovascularization.^[^ ^115^ ^]^ Similiarly, Shiekh et al. used cryogelation technology to prepare a PUAO−CPO cryogel by incorporating CPO into a PUAO scaffold, reducing ROS and releasing O_2_ continuously for 10 days.^[^ ^116^ ^]^ This material addressed both excess ROS and the reduction of angiogenesis due to local hypoxia. Several studies showed that metallic and nonmetallic nanoparticles could both reduce the formation of ROS and boost ECs' migration and incipient tube formation, such as zinc oxide,^[^ ^117^ ^]^ titanium oxide,^[^ ^118^ ^]^ lanthanide metallide,^[^ ^119^ ^]^ silver,^[^ ^120^ ^]^ gold,^[^ ^121^ ^]^ graphene oxide,^[^ ^122^ ^]^ and carbon nanotube.^[^ ^123^ ^]^

**Figure 5:**
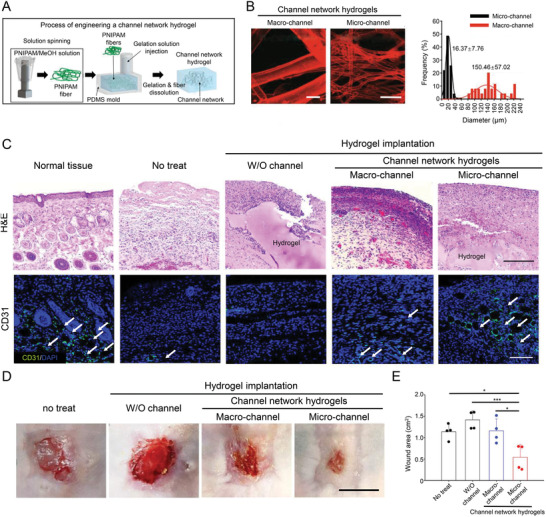
Micro‐channel network hydrogels induced ischemic blood perfusion connection. A) Schematic illustration of procedure to produce PNIPAM fiber, channel network in a hydrogel within a PDMS mold. B) Confocal visualization of micro‐ or macro‐channel networks in hydrogels with their channel diameter distribution. Scale bar = 100 µm. C) Representative images of general histology (H&E) and CD31^+^ cells (green) with nuclei (blue DAPI). White arrows point out microvascular structures (CD31^+^) in the skin tissue sections. Scale bar = 100 µm. D) Photographs of wound healing sites on day 14 postimplantation. Scale bar = 1 cm. E) Degree of decreased wound area from the initial 2 × 2 cm defect in each group on day 14 post‐implantation (*n* = 4). Reproduced with permission.^[^ ^126^ ^]^ Copyright 2020, Springer Nature. H&E, hematoxylin and eosin; PDMS, polydimethylsiloxane; PNIPAM, poly(*N*‐isopropyl acrylamide); W/O, without.

Bioglass and mesoporous silica nanospheres fabricated on nanofibrous membranes have the potential to release more silicon ion and upregulate the expression of genes associated with angiogenesis and new tissue formation.^[^ ^100^ ^]^ A hydrogel based on fayalite and *N*,*O*‐carboxymethyl chitosan (NOCS) was heated in situ by the photothermal effect, mimicking the “hot spring effect” and bound to the released bioactive ions (Fe^2+^ and SiO_4_^4−^). This stimulated the secretion of GFs to promote angiogenesis and may activate the HSP90/eNOS pathway and bFGF/bFGFR signaling in endothelial cells.^[^ ^124^ ^]^ Similarly, Wu et al. presented a redox‐regulated, miRNA‐impregnated ceria nanozyme‐enhanced self‐protective hydrogel (PCN‐miR/Col) to remodel the oxidative wound microenvironment into a regenerative one. Meanwhile, the structural integrity of encapsulated proangiogenic miRNAs remained intact. This ensured typical functional neovascularization and SpO_2_ in diabetic ulcer wounds.^[^ ^125^ ^]^ This “seed and soil” strategy concept is valuable in guiding the design of bioactive materials to explore the potential pathological mechanisms of wound healing and solve the puzzle further in regulating the microenvironments to manipulate cell behaviors. Lee et al. embedded solution‐spun poly(*N*‐isopropyl acrylamide) (PNIPAM) fibers in polydimethylsiloxane (PDMS) mold, then eluted PNIPAM to obtain 3D channel network hydrogels. The inward growth of adjacent host vessels connects with the perfusion of micro‐channels to form vascular perfusion throughout the hydrogel implant (**Figure** **5**).^[^ ^126^ ^]^

Nanoparticles can promote angiogenesis by being endocytosed into ECs or immune cells by caveolae and clathrin, inducing altered cell behaviors.^[^ ^127^ ^]^ Paterson et al. found that in the concentration range of 30−300 ng mL^−1^, the copper‐containing mesoporous glass nanoparticle demonstrated its capacity to promote EC proliferation along with the function of angiogenesis tested by applying aortic rings and chick chorioallantoic membrane assays.^[^ ^128^ ^]^ Combining microbubble therapy with tissue engineering has recently become an infusive new endeavor, where the use of microbubble with hydrogel can overcome the limitation of O_2_ diffusion and provide rapid and advanced wound healing.^[^ ^129^ ^]^

Apart from ECs, another type of cells known as pericytes are necessary for angiogenesis. Pericytes surround vascular ECs and perform functions, such as creating a vascular barrier to bacteria, regulating blood flow, and disrupting and stabilizing the microvascular system.^[^ ^2^ ^]^ However, relatively few studies have stressed the effects of bioactive materials on pericytes. The interactions between ECs and pericytes have yet to be explored. The following step must explore questions related to the individual development and origin of pericytes, enabling the full exploitation of their properties to develop more effective bioactive materials to facilitate angiogenesis.

#### 2.3.3 Granulation Tissue Formation

Fibroblasts were previously thought to be the only source of granulation tissue, stimulated by various growth signals to proliferate and migrate with some transforming into myofibroblasts after exposure to mechanical signals and GFs, such as TGF‐*β*. However, some of the fibroblasts in granulation tissue are derived from bone marrow‐derived mesenchymal stem cells (BM‐MSCs).^[^ ^5^ ^]^ The different concepts offer the opportunity to develop bioactive materials to regulate fibroblast behaviors. For example, the dual drug‐loaded bilayer nanofibrous sponge‐like 3D scaffold fabricated from keratin‐fibrin‐gelatin‐mupirocin 3D sponge with electrospinning fiber of poly(3‐hydroxybutyric acid) and gelatin loaded with curcumin were shown to promote fibroblast migration and potentiate Col synthesis by imitating the features of the ECMs. The main component of the secreted Col was revealed to be hydroxyproline, which acted as a significant player in the ECM deposition during wound healing.^[^ ^130^ ^]^ Due to the unique cell‐recognition motifs (RGD), the nonmulberry SF reinforced the cell−material interaction, resulting in increased recruitment and adhesion of keratinocytes and fibroblasts, which accelerated the granulation formation.^[^ ^131^ ^]^

Granulation tissue inhibits bacterial invasion, acts as a scaffold for cell proliferation and migration, and is particularly important for tissue remodeling. The granulation thickness is often considered as an indicator of the degree of wound healing. However, more interest has been devoted to modulating the performance of fibroblasts during matrix remodeling and scar formation. This phase might be less emphasized individually because of its overlap with angiogenesis and matrix remodeling.

### 2.4 Promotion of Matrix Remodeling by Control of Cell Expression

The utilization of bioactive materials to modulate the matrix remodeling phase of a wound is broadly based on two ideas. The first is to promote wound contraction and matrix deposition in the early stages, and the second is to alleviate the inflammatory response and limit the excessive proliferation of myofibroblasts and myeloid cells in the later stages to reduce scar formation.

The remodeling phase of wound healing occurs at the end of the proliferation phase during which keratinocytes are involved in wound reepithelialization, while fibroblasts and ECs deposit the ECMs.^[^ ^132^ ^]^ Modulating the behaviors of fibroblasts and myofibroblasts by bioactive materials significantly facilitates wound contraction and closure in the early stage of matrix remodeling, as the contribution of (myo)fibroblasts to this phase is prominent. Watarai et al. designed a starPEG−heparin hydrogel introducing RGD peptides to achieve the sustained release of TGF‐*β* to induce fibroblast differentiation into myofibroblast, thus improving the expression of ED‐A fibronectin, Col I, and incorporating *α*‐SMA and palladin into F‐actin stress fiber.^[^ ^133^ ^]^ A study showed that SF hydrogel has a higher potential to promote wound healing in comparison to Col gel and induces the expression of TNF‐*α* and CD163, indicating a shift from inflammation to the proliferative remodeling phase of healing progress.^[^ ^134^ ^]^

**Figure 6:**
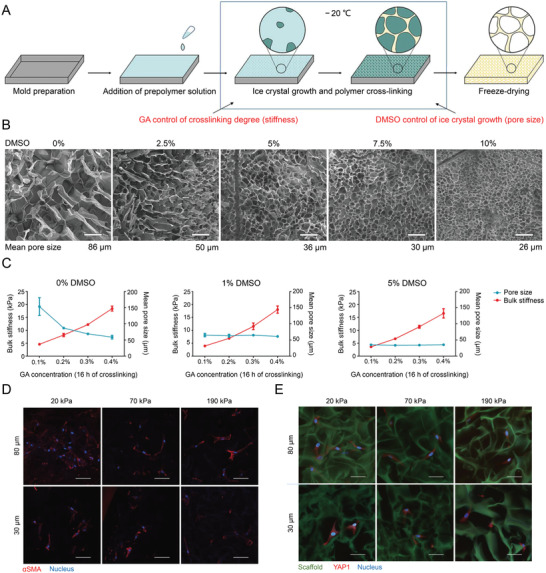
Cryoprotectant enables structural control of porous scaffolds for the exploration of 3D cellular mechano‐responsiveness. A) Schematic illustration of scaffold fabrication via cryogelation of prepolymer solution in presence of cryoprotectant for pore size control and chemical cross‐linker for stiffness control. B) SEM images of gelatin scaffolds cross‐linked by GA demonstrating correlation of pore size and DMSO concentration. Scale bar = 100 µm. C) Traditional stiffness control using varied concentrations of chemical cross‐linker (GA) caused a change in scaffold pore size (stiffness, *n* = 3; pore size, *n* = 5). With the introduction of 1% or 5% DMSO, the pore size remained unchanged at 30 or 60 µm, respectively. D) Significant *α‐*SMA expression with fiber‐like accumulation was only observed in the scaffold with larger and stiffer pores (80 µm and 190 kPa). Scale bar = 50 µm. E) Images of YAP1 showed the significant cytoplasmic localization in small pore scaffold and nucleus localization in 80 µm and 190 kPa  group. Scale bar = 50 µm. Reproduced with permission.^[^ ^137^ ^]^ Copyright 2019, Springer Nature. DMSO, dimethylsulfoxide; GA, glutaraldehyde; SEM, scanning electron microscope.

For epithelial and fibroblast cells, the contribution of substrate stiffness to cell traction has been quantified, and thus the cell movement can be regulated by altering substrate stiffness by activating myosin II.^[^ ^80^, ^135^ ^]^ YAP is a well‐known mechanotransduction transcription factor whose nuclear localization activates F‐actin‐stained human dermal fibroblasts (HDFs) in 3D porous scaffolds and upregulates the expression of *α*‐SMA in fibroblasts.^[^ ^136^ ^]^ A previous study achieved individual control of the scaffold pore size and stiffness by using a cryoprotectant. The fibroblasts achieved maximal activation and displayed the highest YAP nuclear‐to‐cytoplasm ratio on scaffold with the highest pore size and stiffness (80 µm and 190 kPa). The expression pattern of Col I was likewise similar to that of YAP (**Figure** **6**).^[^ ^137^ ^]^

ECM remodeling must be tightly controlled to establish a normal wound repair process. MMPs can degrade specific components of the ECMs, thus slowing matrix synthesis, which is a critical step in remodeling.^[^ ^138^ ^]^ After modifying ECMs, TIMPs inhibit the function of MMPs, preventing the continuous degradation of ECMs.^[^ ^139^ ^]^ Thus, designing bioactive materials for the proper regulation of MMP/TIMP is a promising option. Fortunately, sulfated GAGs were found to bind to TIMP‐3 specifically, prolonging its beneficial existence in the wound site without affecting its ability to inhibit the proteases MMP‐1 and ‐2. Thus, bioactive materials containing sulfated GAGs can delay the process of matrix degradation and shift the MMP/TIMP equilibrium toward a healing phenotype.^[^ ^140^ ^]^

Another important feature during the ECM remodeling is the control of cell suicide‐apoptosis of myofibroblasts and inflammatory cells, thereby inhibiting and terminating the healing process.^[^ ^21b^ ^]^ Myofibroblasts require prompt elimination to avoid the overproduction of ECMs and scarring caused by over‐contraction of the wounded tissue. Furthermore, chronic activation of anti‐inflammatory macrophages will lead to fibroblast hyperactivity, resulting in the excessive production and deposition of ECM proteins.^[^ ^69^ ^]^ As mentioned earlier, TGF‐*β* may result in the development of fibrosis. Thus, a silk‐fibroin/gelatin electrospun nanofibrous dressing, functionalized by astragaloside IV, was previously shown to prevent full‐thickness scar formation by reducing TGF‐*β*1 secretion and Col I/III ratios.^[^ ^141^ ^]^

According to recent reports, the accumulation of matrix components and increased tissue stiffness could shift macrophages toward a wound‐healing phenotype.^[^ ^58^ ^]^ In the late stage of tissue remodeling, the role of macrophages becomes essential. IL‐10 secreted by macrophages and CD4^+^ Th lymphocytes can de‐differentiate myofibroblasts into fibroblasts.^[^ ^142^ ^]^ If (myo)fibroblasts overexpress CD47, they will be blocked from being phagocytosed and cleared by macrophages.^[^ ^143^ ^]^ Chen et al. constructed an “Inner−Outer” electrospun fiber that released a low concentration of IL‐10 within the wound to promote the polarization of macrophages toward the M2c phenotype, ultimately contributing to scarless skin regeneration.^[^ ^144^ ^]^ As CD4^+^ Th lymphocytes induce macrophage polarization, they can also inhibit neutrophil and macrophage infiltration and Col deposition during wound healing and increase microvascular formation.^[^ ^145^ ^]^

Hence, the crosstalk between fibroblasts and immune cells will be an indication for us to design bioactive materials that promote the regeneration process during wound healing. Bioglass has substantial effects on the behaviors related to the gap junction of ECs and Cx43 gap junction channels. It further promotes M2 macrophage polarization at the site of cutaneous trauma, inhibits the inflammation response, and modulates the paracrine effect between macrophages and surrounding cells.^[^ ^146^ ^]^ Ma et al. designed injectable sodium alginate/bioglass (SA/BG) composite hydrogel loaded with SA particle containing conditioned medium (SA_CM_), where PLGA microsphere was encapsulated with pirfenidone (SA/BG‐SA_CM_‐PLGA_PFD_). This represented a sequential delivery system that improved therapeutic efficiency by addressing three challenges: regulating the inflammatory response, promoting the formation of vascularized granulation tissue, and preventing the fibrosis and scarring of regenerative skin.^[^ ^147^ ^]^ It is discovered that high molecular weight hyaluronan (HMW HA) could increase phagocytosis and IL‐10 expression by directing M1 to M2 resident macrophage polarization.^[^ ^148^ ^]^ Surprisingly, when HMW HA was added to lipopolysaccharide (LPS)‐activated M1 macrophages, the M1 phenotype could even be reversed, decreasing the transcription of pro‐inflammatory genes, *nos2* and *il12b*, and increasing IL‐10 level at the same time.^[^ ^149^ ^]^ Recently, a remarkable increase of the colony‐stimulating factor‐1 receptor (CSF1R) expression was observed after the implantation of various bioactive materials. By inhibiting CSF1R, fibrosis was eliminated, while other functions of the macrophage were preserved. This result suggested that targeting CSF1R may be an approach to inhibit fibrosis and improve the biocompatibility of bioactive materials without extensive immunosuppression.^[^ ^150^ ^]^

With the increasing popularity of stem cell therapy, the combination of bioactive materials with mesenchymal stem cells has aroused significant interest. A series of experiments demonstrated that hydrogels combined with human umbilical cord mesenchymal stem cells (hUC‐MSCs), adipose‐derived mesenchymal stem cells (A‐MSCs), or BM‐MSCs accelerate tissue remodeling and inhibit scar formation on severe wounds by promoting Col deposition as well as the secretion of bFGF and TGF‐*β*1 by ECs, phagocytes, etc.^[^ ^151^ ^]^ Zomer et al. combined dermal‐derived MSCs (D‐MSCs) and A‐MSCs with a commercial Col‐based scaffold, Integra, and applied them to full‐layer wounds in mice, indicating that they were also capable of modulating macrophage polarization. The therapeutic effect of both combinations was revealed to be superior to that of Integra alone.^[^ ^152^ ^]^

## 3 Challenges and Perspectives

Currently, for most bioactive materials, the explorations of mechanisms and the validations of therapeutic effects are obtained in vitro and in vivo. The challenges of extrapolating these outcomes to human patients are significant limitations for the clinical translation of new therapies.^[^ ^71^ ^]^ The human immune system is highly evolved and differs significantly from that of animals. The phenomena and effects observed in animal models may not be fully applicable to humans. As introduced in Section 2.2, FBR is also a major obstacle to applying bioactive materials. Although numerous studies targeted modification techniques to alleviate the development of FBR, there are some significant differences between the inflammatory response in murine and humans and even between macrophage behaviors.^[^ ^153^ ^]^ For example, nitric oxide and arginase, the most commonly used markers for detecting M1/M2 macrophage polarization in murine, are rarely expressed in humans.^[^ ^154^ ^]^ Moreover, the evaluation and application of the bioactive materials must also consider the specificity of the patients' conditions. The individual differences in human diseases make it challenging for bioactive materials to achieve targeted treatment, thus impeding the promotion of their clinical application.^[^ ^155^ ^]^

The accurate manipulation of biological response between bioactive materials and target cells under complex physiological or pathological conditions will be the subject of future studies. There are only a few studies conducted on the involvement of bioactive materials in the induction of stem cell‐directed differentiation, while the sophisticated microenvironmental effects in vivo likewise render the experimental results inconclusive. The results obtained thus far are primarily empirical summaries without further systematic or standardized conclusions, and relevant research currently remains at a preliminary stage. Future research must combine bioactive materials with cutting‐edge technologies, such as single‐cell technology and computer‐aided design, to decipher specific behaviors of various types of cells during the wound healing process to obtain efficient bioactive materials that precisely regulate the microenvironments and cell behaviors.^[^ ^69^, ^156^ ^]^

## 4 Conclusion

Alterations of the wound site microenvironments, including ECMs, cytokines, chemokines, O_2_ levels, mechanical forces, and so forth, directly impact cell activation and recruitment. During the wound healing process, cellular interactions are essential for complete closure, whereby any unbalanced cellular or molecular mechanism may transform a wound into a fibrotic or non‐healing one. Elucidation of the cellular and molecular mechanisms of wound healing lay the cornerstones for developing innovative and effective therapeutic strategies. Knowledge of the differences between various wounds will help improve existing scientific methods and increase wound healing rates. The bioactive materials are broadly defined as a kind of substances, natural or artificial, designed to interact with or regulate biological systems. In recent years, it has received considerable attention owing to its powerful features. The development of bioactive materials offers more advanced and promising treatment strategies. Armed with the information on wound healing, the chemical and physical properties of bioactive materials may be adjusted, such as stiffness, geometric shape, wettability, surface charge, specific functional groups, as well as the bioactive substances they can carry. Consequently, under ideal conditions, we will be able to tailor cell behaviors to improve the speed and quality of wound healing.

## Conflict of Interest

The authors declare no conflict of interest.
